# A clinical study of transcranial ultrasound as an adjuvant therapy for progressive cerebral infarction

**DOI:** 10.3892/etm.2013.965

**Published:** 2013-02-19

**Authors:** XIUJU GAO

**Affiliations:** Department of Neurology, The First Affiliated Hospital, Henan University of Science and Technology, Luoyang 471003, P.R. China

**Keywords:** transcranial ultrasound thrombolysis, progressive cerebral infarction, urokinase

## Abstract

The present study aimed to investigate the clinical efficacy of transcranial ultrasound as an adjuvant therapy in combination with small doses of urokinase (UK) for the treatment of progressive cerebral infarction. Sixty-one eligible patients with progressive cerebral infarction were successively and randomly assigned into one of the following groups; 30 patients to the treatment group (transcranial ultrasound + small doses of UK) and 31 patients to the control group (single small doses of UK). Based on conventional therapy, patients in the treatment group received transcranial ultrasound. The neural function deficit scale and curative effect scores of the two groups were recorded before treatment and on the 7th and 14th days after treatment. No differences in the neural function deficit scale between the two groups was observed before treatment, however, on the 7th and 14th days after treatment, a significant decrease was evident in the treatment group (P<0.01). The overall response rate was 100% in the treatment group and 74.2% in the control group, with a significant difference (P<0.01). Transcranial ultrasound is able to contribute to the thrombolytic effects of UK and prevent the progression of thrombi, subsequently aiding the recovery of neural functions.

## Introduction

Progressive ischemic stroke refers to the symptoms of neurological deficit present after ischemic stroke which demonstrate progressive aggravation and continue until further severe neurological deficits appear within 48 h (1). Progressive ischemic stroke accounts for 30% of all incidences of stroke and is associated with an increased rate of morbidity and mortality. Due to the difficulty in treating such cerebrovascular diseases (2), much attention has been devoted to this group of diseases by clinicians. In this study, we report 30 cases of clinical observation and analysis of transcranial ultrasound combined with small doses of urokinase (UK) for the treatment of progressive cerebral infarction.

## Materials and methods

### Clinical data

Sixty-one patients from The First Affiliated Hospital, Henan University of Science and Technology (Luoyang, China) were enrolled between October 2005 and October 2010. Each patient’s diagnostic criteria met with the international diagnostic criteria of cerebral stroke (3). Patients were successively and randomly assigned into one of the following groups; 30 patients to the treatment group (small doses of UK + transcranial ultrasound) and 31 patients to the control group (single small doses of UK). No significant differences in age, onset time, complications and degree of nervous functional defects were observed between the two groups (P>0.05). Eligibility criteria for patients included: i) aged 40–75 years old; ii) clinical features were in accordance with internal carotid artery system cerebral infarction; iii) onset time was <72 h and the primary stroke symptoms of the nervous system and signs displayed continued progressive aggravation under doctor supervision and medical intervention; iv) cerebral hemorrhage was ruled out by cerebral CT/MRI; v) patients and/or their families provided written informed consent. This study was conducted in accordance with the declaration of Helsinki. This study was conducted with approval from the Ethics Committee of The First Affiliated Hospital, Henan University of Science and Technology.

### Treatment

In the treatment group, 100 ml UK (250,000 units) + physiological saline was intravenously administered within 30 min. Simultaneously, transcranial ultrasound therapy combined with ultrasound through the transtemporal window of the lesion side was performed once a day for 20 min, for a total of 5 days. In the control group, 100 ml UK (250,000 units) + physiological saline was administered, and at the same time a therapeutic instrument was worn without any sound device, with the same treatment regimen as the treatment group. Both groups of patients were routinely administered drugs to promote blood circulation and remove blood stasis (such as Xuesaitong or Shuxuetong) and edaravone, a cerebral protective agent. Patients complicated with diabetes, hypertension or hyperlipidemia were administered the relevant treatment.

### Observation

The two groups of patients routinely underwent cerebral CT/MRI, as well as examination of hemagglutination, blood routine, blood glucose, hepatonephric function and electrolyte levels before and after treatment. Patients were evaluated using the neural function deficit scale before treatment, and on the 7th and 14th days after treatment by neurologists.

### Evaluation of efficacy

According to the latest diagnostic criteria for cerebral stroke modified in 2012, the degrees of clinical neurological deficit and the qualities of lives within each group of patients were scored and their efficacies determined (4): i) almost cured: the deficit score was decreased by 91–100%, with a grade 0 disability; ii) significantly improved: the deficit score was decreased by 46–90%, with a grade 1–3 disability; iii) improved: the deficit score was decreased by 18–45%, with independent life; iv) invalid: the deficit score was decreased by <17%; v) worse: the deficit score was increased by >18% or mortality.

### Statistical analysis

The SPSS 10.0 software was used to analyze the data. The measurement data were measured by χ^2^ inspection and are presented as mean ± SD.

## Results

### Neural function deficit scale

There was no significant difference in the neural function deficit scale between the two groups before treatment, however, a significant difference was evident after treatment. The Scandinavian Stroke Scale (SSS) rating decreased significantly on the 7th and 14th days after administration of treatment within the treatment group, with a significant difference before treatment compared with the control group (P<0.01, Table I).

### Comparison of clinical efficacy

The overall response rate was 100% in the treatment group and 74.2% in the control group, with a significant difference (P<0.01, Table II).

## Discussion

Progressive cerebral stroke is referred to as the increase of neurological deficit within several hours of the onset of disease, generally caused by the amplification of arterial thrombosis, with the exception of hematoma and edema (5). Around one-third of patients with stroke develop progressive stroke (6), which carries a significantly enhanced mortality and disability rate (7), causing an increasing number of difficulties in diagnosis and treatment and doctor-patient disputes. Hemodynamic pathogenesis is characterized by decreased perfusion in the ischemic zone causing collateral circulation insufficiency and progressive circulatory failure, followed by irreversible damage and penumbra expansion in the ischemic penumbra, which leads to the viability of brain cells in this area shortening and becoming unable to transfer information to other cells (8). The number and length of time that these type of cells exist is unstable. These cells are able to evolve into necrotic tissue or normal brain tissue. Therefore, this process may only be prevented after timely rescue of this penumbra (9). In previous years, ultra-early intravenous thrombolysis has achieved significant efficacies for the treatment of acute cerebral infarction (10), however, its time was limited to within 3–6 h. There were different time limits, treatment effects and safety views with respect to the application of UK thrombolysis in treatment of progressive cerebral stroke (11,12). A safe and effective method to extend the time window would prevent further progression in cerebral infarction patients occurring. Reducing the disability rate and improving patients’ quality of life is of great importance to clinicans.

In the present study, transcranial ultrasound therapy was used with an ultrasound instrument with a specific power and frequency (13). In 1976, Professor Lang Hongzhi of our department published the first international article on ultrasonic treatment of cerebrovascular disease (14). Trubestein *et al* reported that ultrasound was capable of destroying and removing intravascular thrombi (15). Subsequent *in vitro* and *in vivo* studies on ultrasound thrombolysis then confirmed that low-frequency ultrasound is able to penetrate the skull and enhance the thrombolytic effect of rtPA, with a good safety profile, which did not damage the blood-brain barrier (16,17). In recent years, a large number of studies have reported that the application of diagnostic transcranial Doppler ultrasonography may enhance the thrombolytic effects of rtPA for the treatment of ischemic cerebral infarction (18). Our department had previously applied UK combined with transcranial ultrasound for the treatment of early acute cerebral infarction and achieved satisfactory results. Transcranial ultrasound with a specific power and frequency is capable of directly acting on the cerebral lesion site through the skull to promote migration of the thrombolytic drug to the blood clot by cleavage of cross-linked fibrin (19), subsequently aiding the thrombolytic effect. The instrument we used previously to perform ultrasound treatment was the type LHZ produced by the Kang Liya (Hong Kong Group) Biological Technology Co., Ltd. (Luoyang, China). It consisted of an ultrasonic generator, a treatment sound head and a fixed head frame. A 0.75-W/cm^2^ pulsed ultrasound was generated with a 3-cm^2^ sound head area and a 800-kHz ultrasonic frequency. The treatment was performed by fixing the sound head on the temporal window of the lesion side and coating the couplant on the surface of the sound head to ensure that the ultrasound penetrated through the skull. During intravenous infusion of UK, the ultrasonic treatment was administered for the same 20 min. *In vitro* tests, animal models and clinical studies have since confirmed that the ultrasound aids a unique biological effect of directly dissolving thrombi and enhancing the effects of thrombolytic drugs (20–22). Its mechanism of action may be summarized as cavitation, micro-flow effect, vibration causing mechanical action, thermal effects, sonochemistry action, ultrasonic penetration action, enhanced fibrinolytic activity, enhanced fiber drugs effect, shortened blood reperfusion time and reversal poisoning (23).

The results of this study demonstrated that the neural function deficit scale of the transcranial ultrasound + small doses of UK group decreased significantly on the 7th and 14th days after treatment compared with the control group (P<0.01). This revealed that transcranial ultrasound contributes to the thrombolysis of UK and may thus be important for the treatment of progressive cerebral infarction by preventing thrombi from progressing and promoting the recovery of neural functions (23). In addition, transcranial ultrasound caused no pain, leading to a high patient compliance, with a quick curative effect and no cell damage caused. Owing to its mechanical effects, ultrasound is able to improve the metabolism levels of brain cells, subsequently aiding the recovery of brain cell function and preventing cell death. Followed by the improvement of brain function (25), the recovery of patient’s paralyzed limb, speech recovery, improved memory, which was worthy of clinical application.

## Figures and Tables

**Table I:** Neural function deficit scale before and after treatment in the two groups (mean ± SD).

Group	n	Before treatment	7 days after treatment	14 days after treatment
Treatment	30	21.01±7.8	15.9±6.21	9.91±8.91^a^
Control	31	20.82±9.53	20.12±9.53	18.3±9.58^b^

Compared with the control group,

a P<0.01; compared with the treatment group,

b P<0.01.

**Table II:** Comparison of efficacy between the two groups.

Group	n	Cases (%)					
		Almost cured	Significantly improved	Improved	No change	Significant efficacy	Overall response rate
Treatment	30	12 (40)	16 (53.3)	2 (6.7)	0 (0)	28 (93.3)^a^	30 (100)^b^
Control	31	5 (16.1)	10 (32.3)	8 (25.8)	8 (25.8)	15 (48.4)	23 (74.2)

Compared with the control group, the significant efficacy

a P<0.05; the overall response rate

b P<0.01.
